# Porcelain Inlays

**Published:** 1904-07

**Authors:** Geo. N. Wasser

**Affiliations:** Cleveland, Ohio.


### PORCELAIN INLAYS.

By Geo. N. Wasser, D. D. S., Cleveland, Ohio.

(Written for *The American Journal of Dental Science.*)

In writing this paper I have nothing new or original to offer. My only excuse for so doing is the hope that it may help those who are interested in porcelain as a filling material.

From an esthetic consideration, it is pre-eminently the ideal filling. The method I will describe is simple, efficient and productive of beautiful results, if every detail is carefully carried out.

Great discretion should be exercised in the use of this material. It is rarely ever indicated back of the six anterior teeth, on account of the inability to obtain sufficient anchorage.

Porcelain restorations involving the incisal edge must be made to avoid all contact with opposing teeth.

### SEPARATION IS IMPERATIVE.

In preparing cavities, undercuts must be studiously avoided and right angles attained as far as possible, that is, in the interior of the cavity. By all means avoid beveled margins. After the cavity is prepared, select your colors, noting the variations in shade from the cervix which is usually a shade darker than the cutting edge. It is understood that the colors must be chosen before the rubber-dam is applied. Where it is possible the rubber-dam should not be used until the inlay is ready to set, as it usually interferes with the procuring of a good matrix. Platinum one one-thousandth of an inch is used for the matrix. This is cut the size desired and held to the cavity, and with pledgets of wet cotton forced to the bottom. Remove the cotton and thoroughly anneal platinum. Carry to place again, and with suitable burnishers (preferably Dr. Reeves' design) it is burnished from within out. Again anneal place in cavity and with English twill tape force to margins holding tightly over tooth and matrix, and thus enabling the operator to burnish matrix without danger of dislodgment. Anneal for the last time and carefully introduce into place, using heavy rubber-dam instead of tape and wetting burnishers to prevent them from sticking to the rubber. In cervical cavities cement impression is used to hold matrix instead of tape and rubber. Each step may be repeated as often as is necessary to secure a matrix free from folds. The matrix is now grasped by the extreme edge with locking pliers, and the foundation body placed therein. In most cases the yellow shade No. 13 is used for the first bake. I have been able to get the best results with Brewster's High Fusing Porcelain.

The powdered porcelain should be mixed with water to such a consistency that it can be readily carved. Keep labial margins free from foundation body allowing sufficient depth to build in selected colors. Bake foundation until slightly glazed, allow to cool, and then build in and carve to desired shape. The building, carving and baking is repeated until the proper contour is accomplished.

Great care should be taken at all times to prevent the porcelain from running over the margins. This can be done by the use of a small camels-hair brush.

After the final baking the filling must be allowed to cool slowly in order to temper it.

When the platinum has been stripped off, bury the face of the inlay in wax, and etch the back with hydrofluoric acid. This usually requires about five minutes. Carefully wash off acid, thoroughly dry, and with rubber-dam in place, the inlay is ready to set. I prefer Ames Special Inlay Cement, which should be used according to directions.

With the cement in cavity, force inlay into place, and hold firmly until cement is set, either with a wooden wedge or a silk ligature tied about tooth and inlay with a surgeon's knot.

---

### SOME REMARKS ON PORCELAIN INLAYS.

By Rea Proctor McGee, M. D., D. D. S., Denver, Colo.

(Written for *The American Journal of Dental Science.*)

